# Supplementary figures and images for: Safety of Biological Therapies in Elderly Inflammatory Bowel Diseases: A Systematic Review and Meta-Analysis
Source: J Clin Med. 2022 Jul 29;11(15):4422. doi: 10.3390/jcm11154422 (PMC9369299; doi:10.3390/jcm11154422)

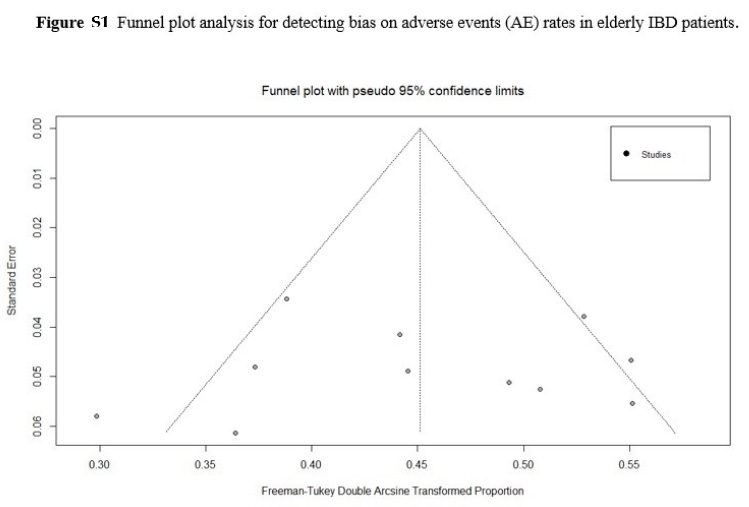

Supplement: Supplementary file 1 [file jcm-11-04422-s001.zip › Supplemental Figure S1 - Funnel Plot on AE rates.jpg]

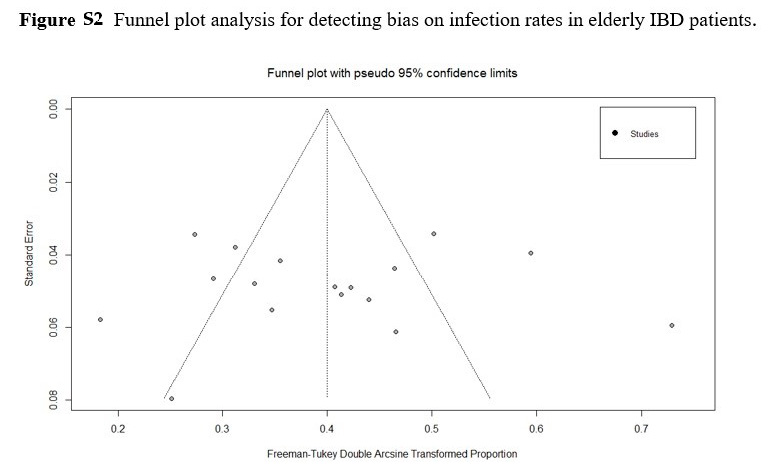

Supplement: Supplementary file 1 [file jcm-11-04422-s001.zip › Supplemental Figure S2 - Funnel Plot on Infection Rates.jpg]

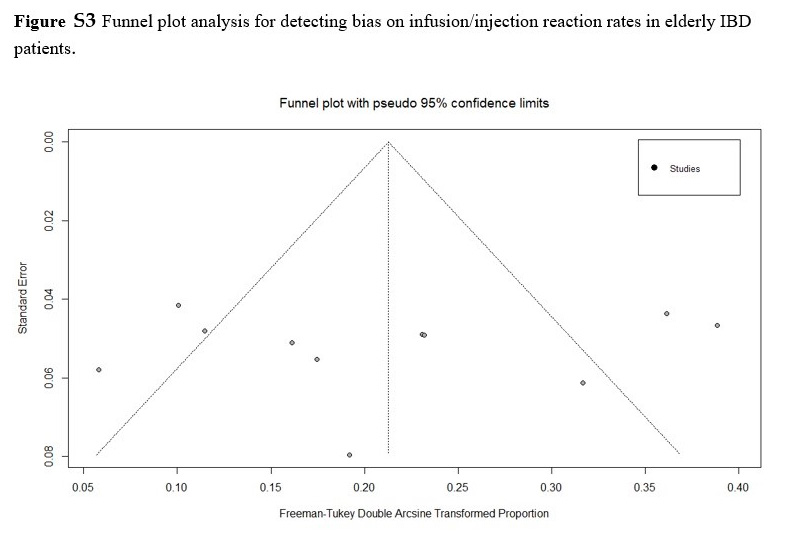

Supplement: Supplementary file 1 [file jcm-11-04422-s001.zip › Supplemental Figure S3 - Funnel Plot on Infusion Reaction.jpg]

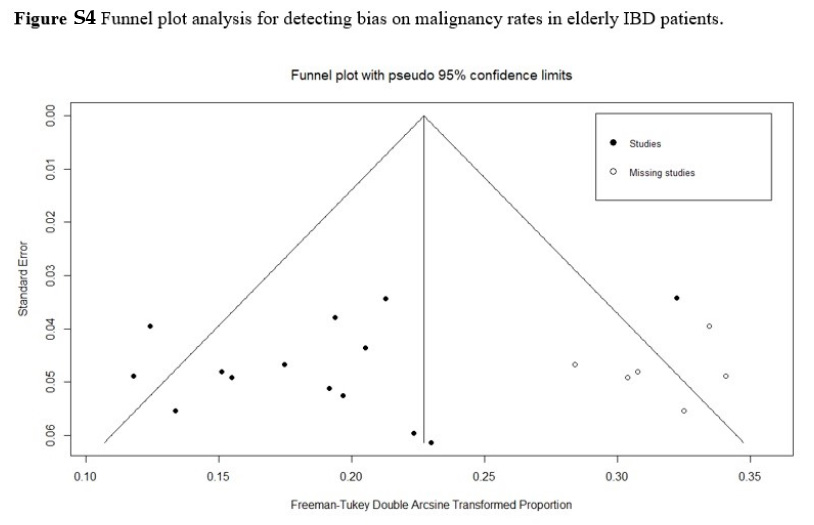

Supplement: Supplementary file 1 [file jcm-11-04422-s001.zip › Supplemental Figure S4 - Funnel Plot on Malignancy Rates.jpg]

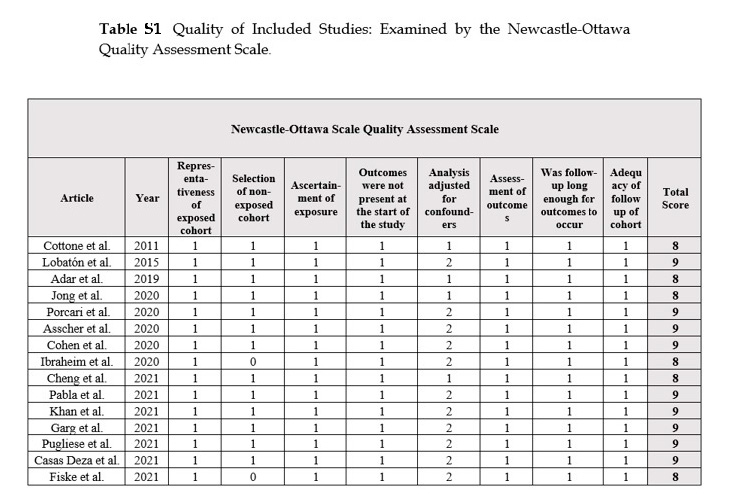

Supplement: Supplementary file 1 [file jcm-11-04422-s001.zip › Supplemental Table S1 - NOS Scale.jpg]

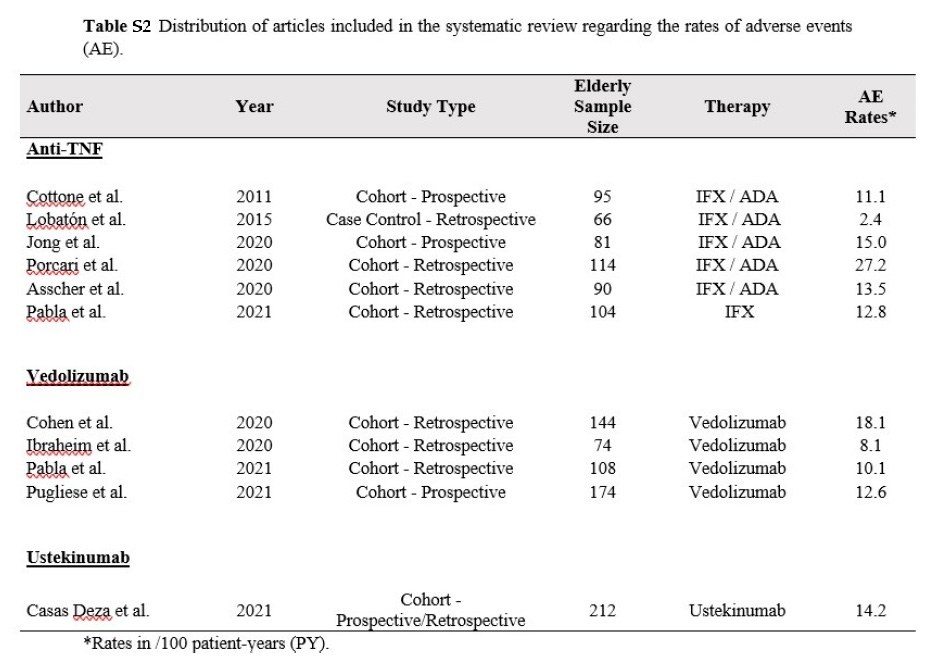

Supplement: Supplementary file 1 [file jcm-11-04422-s001.zip › Supplemental Table S2 - Distribution of Articles (AEs).jpg]

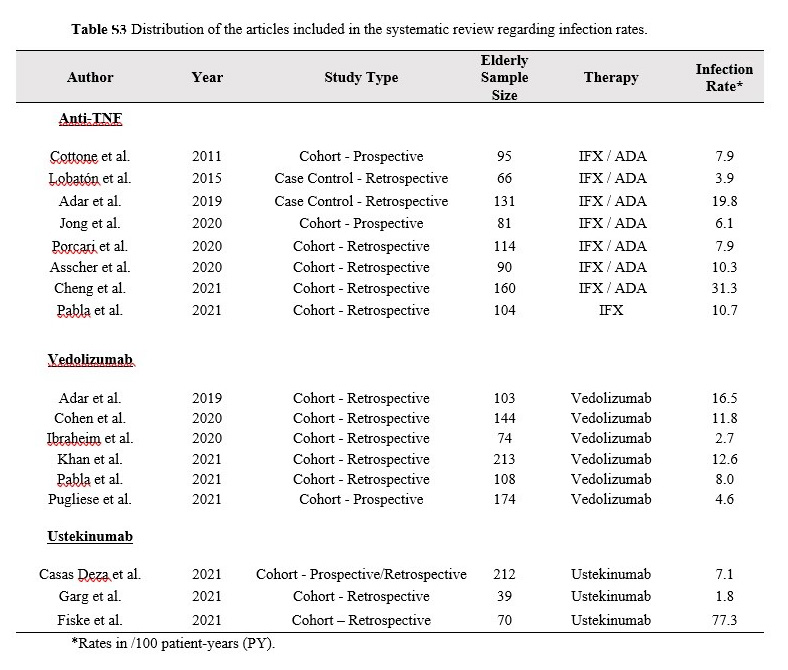

Supplement: Supplementary file 1 [file jcm-11-04422-s001.zip › Supplemental Table S3 - Distribution of Articles (Infection).jpg]

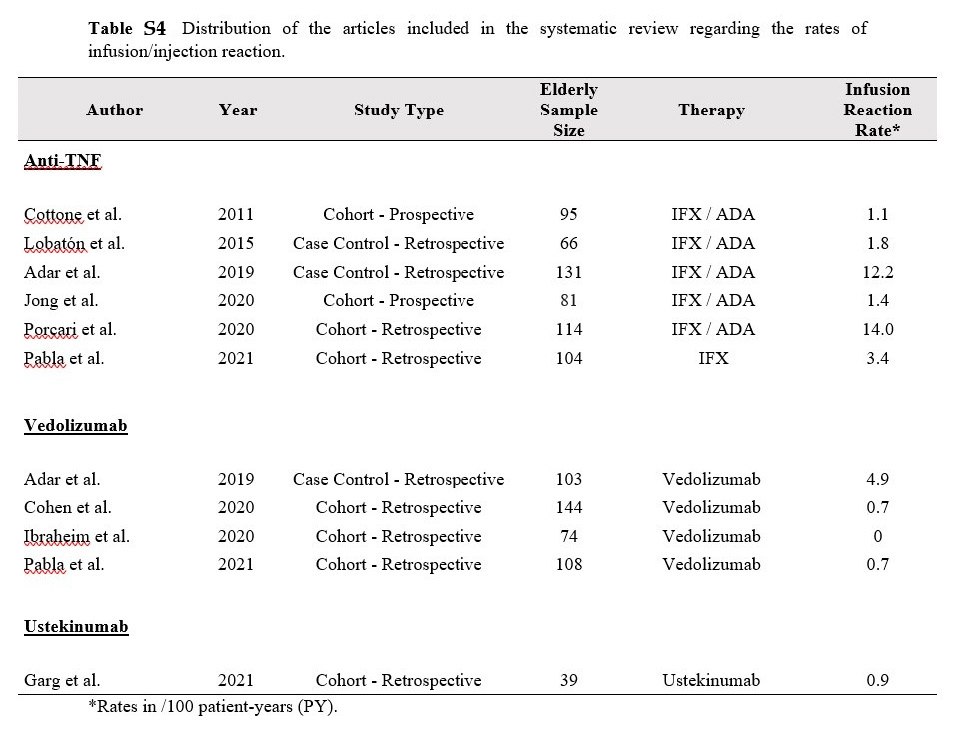

Supplement: Supplementary file 1 [file jcm-11-04422-s001.zip › Supplemental Table S4 - Distribution of Articles (infusion).jpg]

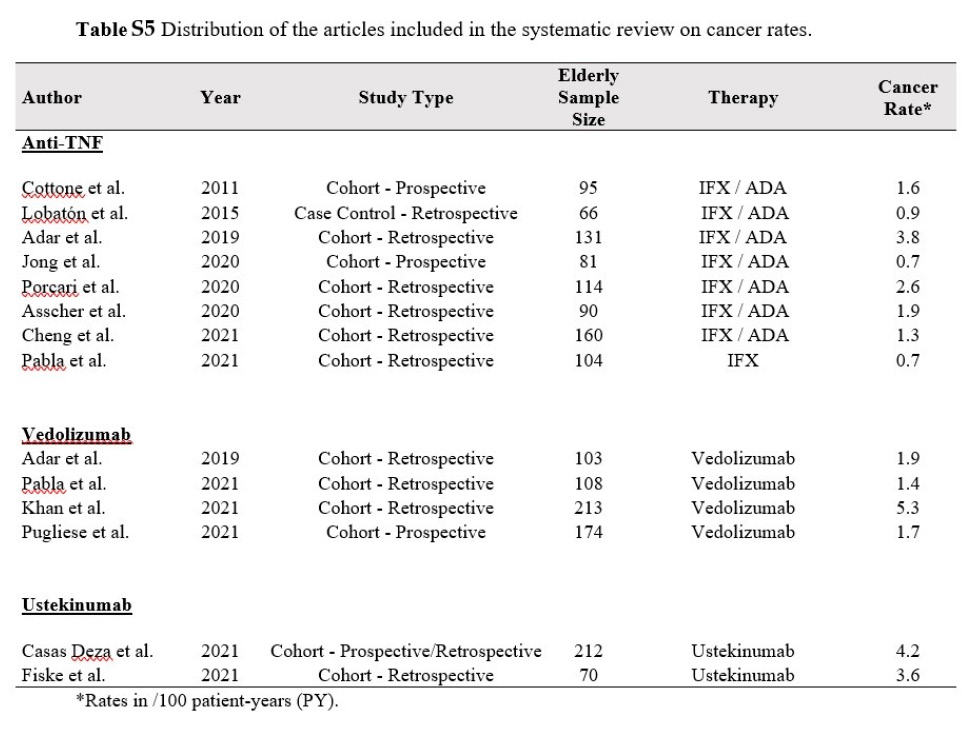

Supplement: Supplementary file 1 [file jcm-11-04422-s001.zip › Supplemental Table S5 - Distribution of Articles (Cancer).jpg]
